# Uncommon southwest swells trigger sea urchin disease outbreaks in Eastern Atlantic archipelagos

**DOI:** 10.1002/ece3.6260

**Published:** 2020-04-24

**Authors:** José Carlos Hernández, Carlos Sangil, Jacob Lorenzo‐Morales

**Affiliations:** ^1^ Departamento de Biología Animal, Edafología y Geología Facultad de Ciencias Universidad de La Laguna San Cristobal de La Laguna Spain; ^2^ Instituto de Enfermedades Tropicales y Salud Pública de Canarias Universidad de La Laguna San Cristobal de La Laguna Spain

**Keywords:** *Diadema africanum*, Eastern Atlantic, mass mortality, *Paramoeba brachiphila*, pathogenic amoebae, sea urchins, Winter storms

## Abstract

Recurrent sea urchin mass mortality has recently affected eastern Atlantic populations of the barren‐forming sea urchin *Diadema africanum*. This new episode of die‐off affords the opportunity to determine common meteorological and oceanographic conditions that may promote disease outbreaks. The population dynamics of this sea urchin species are well known—urchin barrens have persisted for many decades along most of the coastlines off the archipelagos of Madeira, Selvages, and the Canary Islands, where they limit macroalgae biomass growth. However, this new and explosive mortality event decimated the sea urchin population by 93% on Tenerife and La Palma Islands. Two severe episodes of southwestern rough sea that led to winter storms, in February 2010 (Xynthia) and February 2018 (Emma), preceded both mass mortality events. The autumn and winter months of those years were anomalous and characterized by swells with an average wave height above 2 m that hit the south and southwest sides of the islands. The amoeba *Paramoeba brachiphila* was the only pathogen isolated this time from the moribund and dead sea urchins, suggesting that the amoeba was the primary cause of the mortality. This new sea urchin die‐off event supports the “killer‐storm” hypothesis that has been already described for western Atlantic coasts. These anomalous southwest storms during winters generate pronounced underwater sediment movement and large‐scale vertical mixing, detected in local tide gauge, which may promote paramoebiasis. This study presents valuable insights about climate‐mediated changes in disease frequency and its impacts on the future of coastal marine ecosystems in the Atlantic.

## Graphical abstract

Two unusual southwest rough sea periods, with winter storms Xynthia (February 2010) and Emma (February 2018), preceded the sea urchin mass mortality events. This situation suggests that SW storms may promote the outbreak of paramoebiasis episodes in Eastern Atlantic archipelagos, supporting the “killer‐storm” hypothesis.

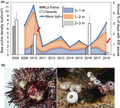

## 1 INTRODUCTION

Sea urchins play a key role in structuring benthic rocky ecosystems in temperate and subtropical regions of the oceans (Harrold & Pearse, 1987; Lawrence, 1975). Their population density often undergoes marked fluctuations that promote a state shift in the ecosystem they inhabit (Hernández, 2017; Ling et al., 2015). Population increases of some sea urchin species can result in catastrophic environmental changes because the animals decimate erect macroalgae cover by grazing. This less‐productive ecosystem state is known as “urchin barren.” Urchin barren is considered to be an undesirable ecosystem state that has clear negative impacts on commercial reef‐based fisheries and local biodiversity (Hernández, 2017). Therefore, there is a need to understand the dynamics of sea urchin fluctuations and the resilience of macroalgae beds. However, the general dynamics of collapse and recovery of these underwater forests remain poorly defined in many regions of the word (Ling et al., 2015). In some regions, sea urchin barrens can be highly persistent and last for many decades (Hernández, 2017; Watanuki et al., 2010). In other regions, there is a cyclical alternation between ecosystem states in which recurrent sea urchin mass mortality helps kelp beds recover every 10–20 years (Scheibling, Feehan, & Lauzon‐Guay, 2013).

Several mass mortality causative agents have been detected in sea urchins, including high temperatures during extreme low tides and different epizootic pathogens and toxicity resulting from harmful algae blooms (Feehan & Scheibling, 2014; Jurgens et al., 2015). Among the epizootic pathogens, amoebas have been identified as a major actor in widespread die‐off events along Northwest Atlantic shores (Feehan & Scheibling, 2014) and recently in Eastern Atlantic archipelagos (Clemente et al., 2014). In the Northwest Atlantic, storm activity and high temperatures have been also well correlated with amoeba blooms (Paramoebiasis) that resulted in sea urchin die‐off (Scheibling et al., 2013). In other regions where pronounced sea urchin mass mortality has been observed, such as the Caribbean, the pathogens were not properly identified (Lessios, 1988). This situation stems from the inherent complication of isolating pathogens and establishing cause–effect relationships. Therefore, every large mortality events offers a unique opportunity to understand these natural phenomena.

In 2014, we reported for the first time a widespread mass mortality event of *Diadema africanum* sea urchin populations in the Eastern Atlantic archipelagos off Madeira and the Canary Islands that took place from October 2009 through April 2010 (Clemente et al., 2014). Despite the disease's spatial heterogeneity, there was an overall 65% reduction in the population compared with the premortality density. The disease mainly affected the southeastern and southwestern coasts of the islands and extended more than 400 km straight into the Eastern Atlantic. Initial laboratory results strongly suggested that *Vibrio alginolyticus* was involved in the disease. However, we could not rule out the possibility of a synergy between isolated species of bacteria and other pathogens. For instance, a study conducted with *D. africanum* specimens collected during the same mortality event revealed the presence of free‐living amoebae in the coelomic fluid of diseased individuals. This amoeba species was identified as *Neoparamoeba branchiphila* (now *Paramoeba branchiphila*; Dyková, Lorenzo‐Morales, Kostka, Valladares, & Pecková, 2011)*.* Confirmed later using small subunits of nuclear rDNA, this species of amoeba was found to be closely related to *Paramoeba invadens* from the western Atlantic region (Feehan, Johnson‐Mackinnon, Scheibling, Lauzon‐Guay, & Simpson, 2013). 

A second sea urchin mass mortality event was recently detected in Eastern Atlantic archipelagos off Madeira and the Canary Islands. This recurrent episode of urchin population die‐off provides evidence for a link with large‐scale meteorological and oceanographic events. We believe that this event can help generalize a natural process to explain sea urchin mass mortality occurring in subtropical and temperate north Atlantic coastal regions. The primary objectives of this study included (a) comparing sea urchin densities before and after the recent mass mortality, using well‐monitored sites; (b) identifying the pathogen with molecular techniques; and (c) performing a long‐term meteorological–oceanographic exploration to determine links between sea urchin population die‐offs and large‐scale oceanographic phenomena, such as storms. Although the idea proposed here is not new and other authors have linked large‐scale meteorological and oceanographic events with sea urchin mortalities (see the review by Feehan & Scheibling, 2014), this new mortality episode, in a different region and affecting a different species, provides evidence to support a general explanation for sea urchin mass die‐offs. In this sense, the findings described here are central to better understanding the dynamics of sea urchin disease outbreaks and the alternation between sea urchin barrens and macroalgae forests. Determining the general mechanisms of this natural event in the East Atlantic Archipelagos will help scientists make accurate predictions of climate‐mediated changes in disease frequency and determine the impacts of this disease on the future of coastal marine ecosystems in the Atlantic.

## 2 MATERIALS AND METHODS

### 2.1 Sea urchin population monitoring

**FIGURE 1:**
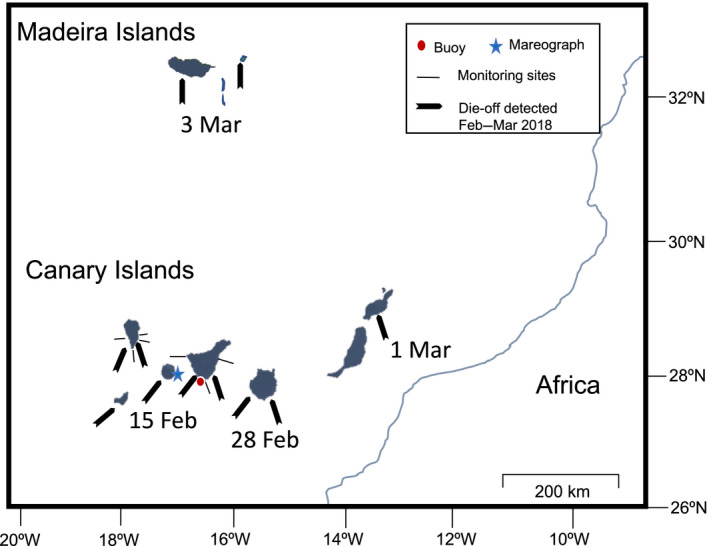
Map showing Madeira and the Canary Islands archipelagos with coordinates. The red circle marks the buoy position and the blue star the mareograph position, the thin black line indicates the monitoring sites in Tenerife and La Palma Islands and the arrow indicates locations, on other islands, where *D. africanum* die‐off was detected by divers

**FIGURE 2:**
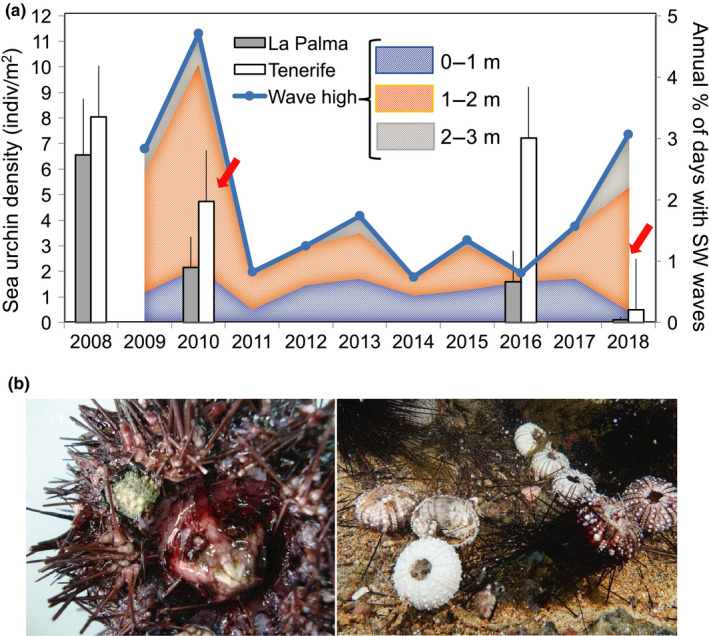
(a) Shaded areas indicate the annual percentage of days with southwesterly waves due to winter storms; colors indicate wave height (see the scale). The bars indicate the mean density ± standard deviation (*SD*) of *Diadema africanum* before and after the mass mortality events on La Palma and Tenerife. Averages were calculated using the Abades, Boca Cangrejo, and Teno site density data for the studied years on Tenerife Island and the Pta. Fraile, Playa del Pozo, Pta. Fuencaliente, and La Bombilla sites on La Palma Island. Red arrows indicate the mass mortality year. (b) *D. africanum* individuals showing first signs of the disease on the ambulacral plates column, and a general view of the Las Galletas site on the southwest coast of Tenerife (Photos: José Carlos Hernández and Antonio Espinosa)

*Diadema africanum* densities have been intermittently monitored at Abades, Boca Cangrejo, and Teno sites, on Tenerife Island, since 2001; and at Pta. Fraile, Playa del Pozo, Pta. Fuencaliente, and La Bombilla, on La Palma, since 2008 (Figure 1). Sea urchins were sampled using 10 × 2‐m transect lines laid randomly over the rocky substrate and parallel to the coastline. A total of 8–10 replicates per site were used to estimate average densities. The sites were characterized by shallow rocky bottoms (0–20 m) dominated by sea urchin barrens (Hernández, Clemente, Sangil, & Brito, 2008). During the mortality episodes, the densities of sea urchins dropped abruptly at all of the sites. In order to quantify the decline in sea urchin densities, we focused on the years 2008–2010 and 2016–2018 (Figure 2a). We selected these years because we had urchin density data for all of the seven sites cited above, which allowed us to perform a more robust before–after comparison. In parallel, all the divers and colleague's die‐off observations were registered, considering the site and the orientation of the island where it was observed (Figure 1). It is important to notice that the densities estimations, during the winter storm years, were done after the first die‐off observations were detected.

### 2.2 Pathogen identification

Soon after the second event started, we collected 15 moribund sea urchins in a very early stage of infection from two localities off the southeastern coast of Tenerife (Figure 2b). The sea urchins were brought to the laboratory of Ciencias Marinas, Universidad de La Laguna, for analysis. Both amoeba and *Vibrio* tests were performed. To isolate amoebae, coelomic fluid (up to 5 ml) was extracted from the sea urchins after draining the seawater using an 18G × 1.5ʹ needle and sterile syringe, which was inserted through the peristomal membrane at an angle that avoided contact with the lantern area (Dyková et al., 2011). A few drops of these samples were cultured on non‐nutrient seawater agar prepared from seawater collected from the same place as the sea urchins. The plates were cultured at 20°C and checked daily for the presence of amoebae. Primary isolate colonies were obtained following this procedure. For the bacteria test, the samples of coelomic fluid were also added to the surface of specific media to isolate *Vibrio* spp. (TCBS agar, Merck), *Aeromonas* spp. (Aeromonas agar, Oxoid), and *Pseudomonas* spp. (Cetrimide agar, Scharlab) and incubated at 37°C for 48 hr. Additionally, we conducted DNA extraction and PCR amplification of amoebic isolates. DNA from cultures identified as positive for amoebae by microscopy was extracted (Reyes‐Batlle et al., 2016) using a Maxwell^®^ 16 Tissue DNA Purification Kit sample cartridge (Promega) and a Maxwell^®^ 16 Instrument (Promega). DNA yield and purity were determined using a NanoDrop 1000 spectrophotometer (Fisher Scientific). PCR amplification of the 18S rDNA gene was carried out using a universal primer pair FLA‐F and FLA‐R (Reyes‐Batlle et al., 2016). The PCR products were sequenced using the MACROGEN sequencing service (Madrid, Spain), and the sequences were aligned using the Mega 5.0 software program (Pennsylvania State University).

### 2.3 Meteorological and oceanographic long‐term study

**FIGURE 3:**
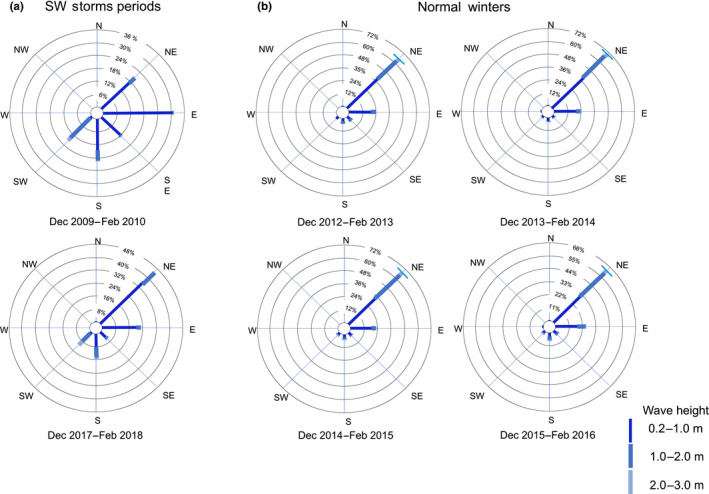
(a) Compass rose showing average wave height and direction from the buoy data for the two winter storm periods: December 2009–February 2010 and December 2017–February 2018. These storm periods were characterized by southwesterly and southern waves with heights of 1–2 and 2–3 m. (b) Compass rose showing average wave height and direction from the buoy data for the winter periods of 2012, 2013, 2014, and 2015. These years were normal and not characterized by winter storms. Most of the days during these study periods were characterized by 0.2–1 m and 1–2 m northeasterly and eastern waves

**FIGURE 4:**
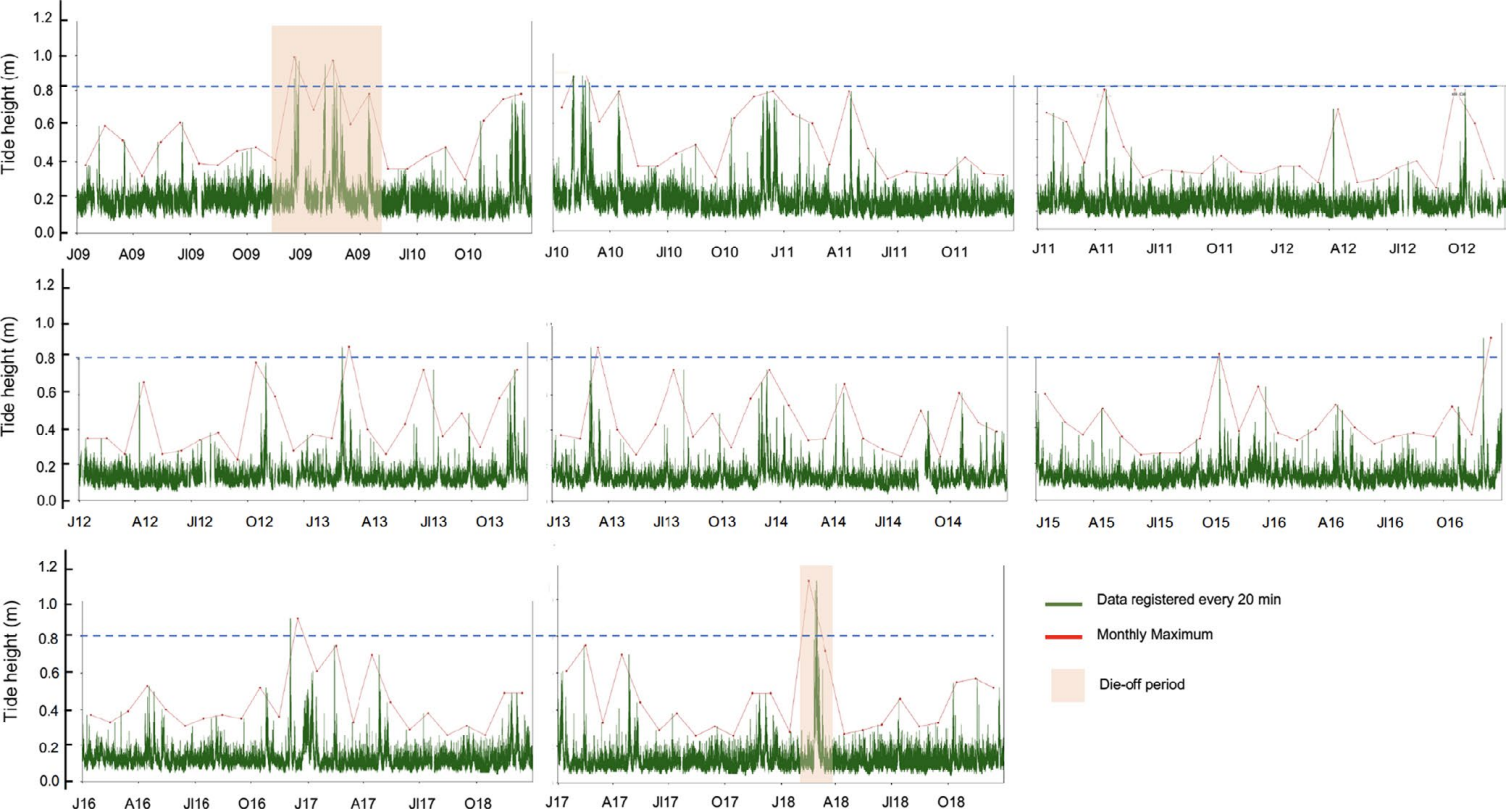
Mareograph data showing the tide height in meters from January 2009 until December 2018. Periods when the die‐off were first registered in the Canaries and Madeira archipelagos are shaded in orange; and it can be seen it coincides with anomalous peaks of tide height registered by the mareograph

In order to determine the combined effects of temperatures and storms on the disease outbreaks that occurred in 2010 and 2018, we analyzed historical meteorological and oceanographic data available since the first mortality event. We focused on 10 years of data from an oceanographic buoy located in Tenerife (Figure 1; data used in Figures 2a and 3), and we also used the high‐resolution tide data from January 2009 until December 2018 period (Figure 4), gathered from the mareograph located in La Gomera Island (Figure 1). Both, the buoy and the mareograph, were located off the southern coast of the Islands where the massive die‐off was first detected. The buoy collected hourly sea surface temperature (SST°C) and the daily wave height and direction data, and the mareograph collected data every 20 min. These data are free accessible from Puertos del Estado‐Ministerio de Fomento, Gobierno de España‐ (http://www.puertos.es/es‐es/oceanografia/Paginas/portus.aspx) as well as the characteristics of the buoy and the mareograph used by Puertos. The buoy wave height and direction data were grouped from December to February to better visualize the winter months over a ten‐year period (Figure 3). The SST°C of every hour was also studied for the entire mortality period from 2010 to 2018, using buoy data.

## 3 RESULTS

### 3.1 Sea urchin population monitoring

In February 2018, we detected another large mass mortality event affecting the Eastern Atlantic archipelagos (Figure 2a). This time, the mortality event was first detected on the western part of the archipelago (Canary Islands), and a few weeks later it spread to Lanzarote, Porto Santo, and Madeira (Claudia Ribeiro and João Canning Clode, personal communication; see Figure 1 for dates), and extending during spring and summer months (Gizzi et al., 2020). On Tenerife, there was an overall average population reduction of 93.2% compared with premortality densities (before 2016 versus after 2018; *F* = 183.81, *p* < .01; Figure 2a). On La Palma island, there was an overall population reduction of 93.1% (before 2016 vs. after 2018; *F* = 55.30, *p* < .01). It is important to note that populations on La Palma never recovered after the first sea urchin mortality event; densities after the event always remained lower than before the first mortality event. For the Madeira archipelago, a rapid recovery after the mortality have been described (Gizzi et al., 2020).

### 3.2 Pathogen identification

The moribund sea urchins exhibited symptoms of the “bald sea urchin disease” described by Clemente et al. (2014) (Figure 2b; Tamura et al., 2011). Species identification was based on sequence homology analysis by comparing the available *Paramoeba* sequences in the GenBank database. No bacterial growth was observed, and all of the infected sea urchins were only positive for *Paramoeba branquiphila*. Despite the detection of this paramoeba, one must be cautious about concluding that the cause of the disease is the amoeba. Additional experiments using Koch's postulate need to be performed.

### 3.3 Meteorological and oceanographic long‐term study

We detected that the mass mortality periods coincided with years when continuous southwest rough seas and a winter storms, named Xynthia (2010) and Emma (2018) by the Institute of Meteorology—Free University of Berlin, hit the archipelagos (Figure 3). The average wave height during these anomalous winters was 2–3 m; the waves came from the south and southwest (Figures 2 and 3). This result was corroborated using wave height and direction average buoy data, from 2009 to 2019, and the satellite images captured daily by the Moderate Resolution Imaging Spectroradiometer (MODIS) on NASA’s Aqua satellite. For comparison purposes, four more normal winter periods were also included in our analysis. During these normal winters, the highest percentage of waves came from the northeast (Figure 3). SST°C average was 20.7°C during the winter of 2010 and 18.2°C during the winter of 2018.

## 4 DISCUSSION

Two unusual southwest rough sea period, with winter storms Xynthia (February 2010) and Emma (February 2018), preceded the mass mortality events. This situation suggests that SW storms may promote the outbreak of Paramoebiasis episodes in Eastern Atlantic archipelagos, supporting the “killer‐storm” hypothesis (Scheibling & Lauzon‐Guay, 2010). No clear correlation with SST°C was found: the first episode occurred with an average SST of 20.7°C and the second occurred with an average SST of 18.2°C. These kinds of rough SW sea with that lead to a winter storms have also been described by meteorologists as “depressions” or “windstorms” and are often associated with strong wind and local precipitation. In the Canary Islands and Madeira, the Azores anticyclone moves toward the northern Atlantic from the end of autumn through mid‐spring and enables the arrival of “normal” northwest storms to the archipelagos. However, when these northwest storms originate at lower latitudes (Liberato et al., 2013), such as Xynthia and Emma, their wet air masses hit the islands from the south or southwest and generate severe windstorms with strong local rain episodes and waves heights above 2 m and storm surge (Figures 3 and 4). These kinds of winter storms develop in the Atlantic, off Madeira, and cross the Canary Islands and then start moving northeast toward the European mainland.

The “killer‐storm” hypothesis was described by Scheibling and Hennigar (1997) and modeled by Scheibling and Lauzon‐Guay (2010). This hypothesis explains the occurrence of recurrent sea urchin mortality events off the coast of Canada as being due to tropical storms and hurricanes (Feehan & Scheibling, 2014; Scheibling et al., 2013). The mechanism involves a tropical storm, a pathogen, and sea urchin populations (Feehan, Scheibling, Brown, & Thompson, 2016; Scheibling & Hennigar, 1997). Thanks to long‐term coastal monitoring and good baseline data of sea urchin populations, Scheibling and Lauzon‐Guay (2010) created a logistic regression model that demonstrated that the probability of mass mortality of sea urchins can be predicted by the intensity and proximity of tropical storms and hurricanes. These tropical storms have been hypothesized to deliver the amoeba *Paramoeba invadens* to the coast, triggering disease outbreaks (Scheibling et al., 2013). Although questions remain about the source populations of the pathogenic agent and the oceanographic mechanisms affecting its introduction to coastal environments, the model of Scheibling and Lauzon‐Guay (2010) has been supported by experiments and field observations (Feehan, Scheibling, & Lauzon‐Guay, 2012; Scheibling et al., 2013).

Our results differ from those of our first study (Clemente et al., 2014) because *Vibrio* was also found in dead urchins at that time. Our hypothesis is that during the first mortality event in 2010, when *Vibrio algynoliticus* and *Parameba braquiphila* were isolated, the sea urchins were probably collected in an advanced stage of infection or when they were already dead. This may also be the case of the recent study performed by Gizzi et al. (2020) in Madeira, who used late infection stage individuals and do not even look for amoebas. However, this second mortality event suggests that the first pathogen to infect the sea urchins could have been an amoeba. Observations of the moribund sea urchins revealed that the amoeba started to damage the epidermis located on the oral side and over the animals’ ambulacral plates (Figure 2b). This pattern of infection could then accelerate the invasion of amoebae through the ambulacral pores as well as facilitate the entrance of other pathogens such as *Vibrio* in later infection stages. Therefore, *Vibrio* and other bacteria could play an opportunistic role while the amoeba may have been the primary cause of sea urchin mortality. These results are also consistent with the findings of studies conducted along western Atlantic coasts that found that paramoebiasis caused sea urchin mass mortality (Feehan & Scheibling, 2014).

We believe that explosive southwest storm events generate pronounced underwater sediment movement and large‐scale vertical mixing (Figure 4). Other authors have also suggested that storm events can transport amoebae via horizontal advection from distant source populations or vertically mix amoebae residing locally in deep basins (Scheibling & Hennigar, 1997, Scheibling et al., 2013, but see Feehan et al., 2016). Amoebae are benthic organisms that live in sandy stable environments (Dykova et al., 2005; Nowak & Archibald, 2018) like the ones found on the southwestern and southeastern sides of the Canary Islands. The massive movement of sediment might increase the number of amoebae in the water column and move them to nearby rocky bottoms where sea urchins reside. Significant sediment deposition over the urchins’ habitat after storms might increase the probability of infection and trigger the mass mortality observed across the archipelagos. Moreover, sediment deposition alone might also cause some damage on the sea urchin epidermis, facilitating the infection. It is important to note that these mass mortalities have been mainly observed in populations living near sandy environments on the southern sides of the islands (Clemente et al., 2014), an observation that supports our hypothesis. Generally, the lee sides of islands are not affected by swell; calm sea weather there favors sediment deposition (Hernández et al., 2008). This situation arises because Atlantic archipelagos are mainly affected by northeastern trade winds, even during winter months, and years with strong southwestern/southern storms are rare (Guijarro, Conde, Campins, Luisa‐Orro, & Picornell, 2015; Figure 3). For instance, in this investigation we only found two large similar episodes over a 10‐year period (Figure 3).

The low frequency and variability of southwestern storms can also help to explain why sea urchin barrens have persisted for decades in the Canaries (Hernández, 2017). However, this situation might change in the coming years. As climate change accelerates, the magnitude and frequency of extreme events is expected to continue to increase in the North Atlantic. A recent review by Pardowitz (2015) covering the North Atlantic and parts of Europe showed that the intensity and frequency of winter storms will increase in these regions over the 21st century. Changes in storm intensity and frequency are consistently identified among multiple model projections. It has been found that the North Atlantic Oscillation (NAO) undergoes fundamental changes in its phase and its shape (Pardowitz, 2015). Consistent with diagnosed changes in storm frequency, the NAO has been found to be shifting toward a more positive phase with its action center shifting toward the northeast. This change may be related to more favorable growth conditions for periods of storminess over the eastern parts of the North Atlantic. In this sense, the disease outbreak described here can be used to reveal the mechanisms of sea urchin massive die‐off and provide predictions about future climate‐mediated changes in disease frequency.

## CONFLICT OF INTERESTS

None declared.

## AUTHOR CONTRIBUTION

**José Carlos Hernández:** Conceptualization (lead); Data curation (equal); Formal analysis (lead); Investigation (equal); Resources (lead); Supervision (equal); Writing‐original draft (lead). **Carlos Sangil:** Data curation (equal); Investigation (supporting); Writing‐review & editing (supporting). **Jacob Lorenzo‐Morales:** Data curation (equal); Formal analysis (equal); Investigation (equal); Resources (equal); Writing‐review & editing (equal). 

## Data Availability

Open Access Data. All the oceanographic data used for the long‐term study can be easily accessible from http://www.puertos.es/es‐es/oceanografia/Paginas/portus.aspx; The sea urchin density data and amoeba sequence have been uploaded to ZENODO and are free accessible using the following DOIs: http://doi.org/10.5281/zenodo.3715422 and http://doi.org/10.5281/zenodo.3716129, respectively.
